# Electrical Stimulation Increases the Secretion of Cardioprotective Extracellular Vesicles from Cardiac Mesenchymal Stem Cells

**DOI:** 10.3390/cells12060875

**Published:** 2023-03-11

**Authors:** Haitao Zhang, Yan Shen, Il-man Kim, Yutao Liu, Jingwen Cai, Adam E. Berman, Kent R. Nilsson, Neal L. Weintraub, Yaoliang Tang

**Affiliations:** 1 Medical College of Georgia, Augusta University, Augusta, GA 30912, USA; 2 Cell Biology and Physiology, School of Medicine, Indiana University, Indianapolis, IN 47405, USA; 3 Medical College of Georgia, Augusta University/University of Georgia Partnership, Athens, GA 30602, USA

**Keywords:** electrical stimulation, intracellular communication, extracellular vesicle, nSMase2, hypoxia

## Abstract

Clinical trials have shown that electric stimulation (ELSM) using either cardiac resynchronization therapy (CRT) or cardiac contractility modulation (CCM) approaches is an effective treatment for patients with moderate to severe heart failure, but the mechanisms are incompletely understood. Extracellular vesicles (EV) produced by cardiac mesenchymal stem cells (C-MSC) have been reported to be cardioprotective through cell-to-cell communication. In this study, we investigated the effects of ELSM stimulation on EV secretion from C-MSCs (C-MSC^ELSM^). We observed enhanced EV-dependent cardioprotection conferred by conditioned medium (CM) from C-MSC^ELSM^ compared to that from non-stimulated control C-MSC (C-MSC^Ctrl^). To investigate the mechanisms of ELSM-stimulated EV secretion, we examined the protein levels of neutral sphingomyelinase 2 (nSMase2), a key enzyme of the endosomal sorting complex required for EV biosynthesis. We detected a time-dependent increase in nSMase2 protein levels in C-MSC^ELSM^ compared to C-MSC^Ctrl^. Knockdown of nSMase2 in C-MSC by siRNA significantly reduced EV secretion in C-MSC^ELSM^ and attenuated the cardioprotective effect of CM from C-MSC^ELSM^ in HL-1 cells. Taken together, our results suggest that ELSM-mediated increases in EV secretion from C-MSC enhance the cardioprotective effects of C-MSC through an EV-dependent mechanism involving nSMase2.

## 1. Introduction

Cardiac mesenchymal stem cells (C-MSCs) reside in the heart and express the early cardiac-specific transcription factor GATA4 and MSC markers, including Sca-1, CD105, and CD140 [1,2,3]. These C-MSCs have cardiac reparative properties via a paracrine mechanism mediated by angiogenic and other factors [1,4]. Extracellular vesicles (EVs) are common membrane-bound vesicles that contain various biomolecules, such as lipids, proteins, and nucleic acids. Exosomes are a specific class of small EVs (sEVs), <200 nm in size, that participate in intercellular communication and can be secreted by MSCs [5]. EVs are produced from cells by exocytosis and are taken up by target cells, thus transmitting biological signals between cells locally or distantly [6]. We previously reported that C-MSC-derived EVs protected the acutely ischemic heart from reperfusion injury [7]. Moreover, transplantation of C-MSC-derived EVs increased cardiomyocyte proliferation and enhanced cardiac angiogenesis in a mouse model of myocardial infarction (MI) [3]. A recent meta-analysis of pre-clinical animal studies using stem cell-derived EVs suggested that they are effective at eliciting cardiac repair post-MI [8].

Landmark trials such as COMPANION have shown that cardiac-resynchronization therapy (CRT) improves cardiac function in patients with advanced chronic heart failure [9]. Several clinical trials, including MIRACLE-ICD (Multicenter InSync ICD Randomized Clinical Evaluation), CARE-HF (Cardiac Resynchronization in Heart Failure), and MADIT-CRT (Multicenter Automatic Defibrillator Implantation Trial-Cardiac Resynchronization Therapy), have demonstrated that CRT improves symptoms and reduces mortality in selected patients [10,11]. While CRT may lead to favorable mechanical effects on contractility, its utility in heart failure may be more related to beneficial effects on cardiac bioenergetics and metabolism [12]. Moreover, not all patients with heart failure benefit from CRT [13]. One factor that clearly distinguishes CRT responders from non-responders is QRS duration. In general, the wider the QRS duration in patients with heart failure, the greater the degree of LV dysfunction and mortality, and the stronger the benefit from CRT. This suggests that aberrant electrical activation may impair cardiac function in heart failure, while CRT could improve outcomes in part by normalizing electrical activation. The Multipoint Pacing (MPP) trial evaluated the safety and efficacy of pacing two left ventricular sites with quadrupole leads in patients with heart failure who had an indication for a CRT-D device, and the results show that MPP is safe and effective for treating heart failure [14]. Compared with conventional biventricular pacing, multipoint pacing significantly improves response and super-responsiveness to CRT and reverse left ventricular remodeling [15]. Recent clinical trials also show that ELSM by cardiac contractility modulation (CCM) provides non-excitatory stimulation to the ventricular myocardium, increasing cardiac contractility without increasing oxygen demand in both animal heart failure models and patients with reduced ejection fraction (EF) [16,17]. How ELSM exerts molecular effects locally, particularly on cell-cell communications, is largely unknown.

Kim S.W. et al. [18] reported that electrical stimulation of cardiac stem cells (CSC) at 1.5 V/1.8 cm with a biphasic square pulse (5 ms) at 5 Hz frequency for 3 h increased CSC survival by increasing AKT and GSK3β phosphorylation and FAK activation/cell adhesion while decreasing caspase-3 cleavage. Interestingly, Yang et al. [19] reported a nano-perforation method to stimulate cells by local and transient ELSM to promote the release of extracellular vesicles carrying transcribed mRNA and targeting peptides, suggesting that ELSM can enhance EV secretion from stimulated cells. However, whether ELSM drives EV secretion in C-MSC, and the putative underlying mechanisms, are unknown.

Neutral sphingomyelinase2 (nSMase2) catalyzes ceramide formation, an essential step in the biogenesis and release of EVs shed from cells under stress conditions, and inhibition of nSMase2 decreased the release of EVs under stress conditions [20,21]. In this study, we investigated the effects of ELSM on EV secretion from C-MSC and quantified the expression of the nSMase2 protein in response to ELSM stimulation. We found that ELSM stimulation increased EV secretion by C-MSC, which protected cardiomyocytes from hypoxia-induced apoptosis in an EV-dependent manner. Our data suggest that nSMase2 is indirectly involved in the modulation of apoptosis in HL-1 cells by regulating the secretion of EVs from ELSM-treated C-MSCs.

## 2. Materials and Methods

### 2.1. Isolation of Mouse C-MSC and Cell Culture

C-MSC were isolated from the hearts of 2-month-old male C57BL/6 mice (Jackson Laboratory, Bar Harbor, ME, USA) using a two-step protocol as described previously [22,23]. Briefly, in step 1, ventricular tissue was minced into 1 mm^3^ size, digested in DMEM medium with 0.1% collagenase IV and 1 U/mL dispase for 20 min, and then cultured in fibronectin/gelatin (0.5 mg fibronectin in 100 mL of 0.1% gelatin) coated 6-well plates until the small round phase-bright cells migrated from the adherent explants and proliferated on the fibroblast layer. In step 2, Sca-1+ cells were enriched from the phase-bright cells by the mouse hematopoietic lineage-depletion cocktail kit (STEMCELL Technologies, Vancouver, Canada), followed by enrichment for Sca-1^+^ cells by magnetic-activated cell sorting (MACS) with Sca-1 magnetic beads (Miltenyi Biotec Inc., Auburn, CA, USA) according to the manufacturer’s protocols. Selected Sca-1 cells were cultured and maintained in complete DMEM medium containing 10% fetal bovine serum, 200 mmol/L L-glutamine, 55 nmol/L β-mercaptoethanol, and 1% MEM nonessential amino acids.

### 2.2. ELSM Treatment and EV Purification

C-MSC were seeded in 6-well plates at a density of 2 × 10^5^ cells/well. After 24 h, the 10% FBS medium was changed to a 5% exosome-depleted FBS medium and cultured for 4 h. Cells were then subjected to ELSM using a cultured-cell pacer system (IonOptix) at a frequency of 0.5 Hz, a pulse width of 5 ms, and a voltage of 1.5 V/1.8 cm for 2, 4, 16, 24, 48, or 72 h. Cells without ELSM were used as controls.

Conditioned media (CM) was collected, centrifuged at 1500 rpm for 10 min to separate the supernatant, and then filtered through a 0.22 μm filter to remove cell debris. The final EV fraction was pelleted after ultracentrifugation at 100,000× *g* for 18 h at 4 °C using a swinging bucket rotor. The EV pellets were then re-suspended in PBS and stored at −80 °C until use. We prepared EV-depleted supernatant by ultracentrifugation of cell supernatants at 100,000× *g* for 18 h at 4 °C using a swinging bucket rotor. Pellets of EVs were discarded, and aliquots of EV-depleted supernatant were collected for downstream experiments.

### 2.3. Electron Microscopy and Zeta Analysis

For transmission electron microscopy (TEM) morphology evaluation, 3 μL of EV pellet was placed on formvar carbon-coated 200-mesh copper electron microscopy grids and incubated for 5 min at room temperature (RT), followed by standard uranyl acetate staining. The grid was washed with three aliquots of PBS and allowed to become semi-dry at room temperature before observation with a transmission electron microscope (JEOL JEM 1230, Peabody, MA, USA).

We measured the size and concentration of EV in CM with nanoparticle tracking analysis (NTA) using ZetaView PMX 110 (Particle Metrix, Meerbusch, Germany) and the corresponding software, ZetaView 8.02.28. The ZetaView system was calibrated using 100 nm polystyrene particles.

### 2.4. Small-Interference RNA (siRNA) Transfection

Mouse C-MSC in 6-well plates were incubated with 40 nM of mouse nSMase2 siRNA or scrambled negative control (NC) siRNA (Santa Cruz Biotechnology, Santa Cruz, CA, USA) with Lipofectamine RNAiMax transfection reagent (Thermo Fisher, Waltham, MA, USA) in 2 mL medium for 48 h at 37 °C.

### 2.5. Assessment of Acetylcholinesterase (AchE) Activity

The concentration of EVs was quantified by measuring the activity of AchE (a typical EV marker) using the Amplex^®^ Red Acetylcholine/Acetylcholinesterase Assay Kit (Thermo Fisher) as previously described [23]. Briefly, 10 µL EV fractions were suspended in 90 µL of PBS. Then, 100 μL of diluted EVs were added to each well of a 96-well flat-bottomed microplate. Next, the EV samples were mixed with 100 μL of the working solution, including Amplex Red reagent containing HRP, choline oxidase, and acetylcholinesterase. AchE activity was measured using the GloMax Discover System (Promega, Madison, WI, USA) after incubation in the dark at room temperature for 30 min. For each well, the relative AChE activity was calculated by subtracting background fluorescence, normalizing to protein concentration, and then normalizing to the Ctrl si-NC group.

### 2.6. Isolation and Quantification of mRNA

Total RNA was extracted with RNAzol RT (Molecular Research Center, Inc., Cincinnati, OH) according to the manufacturer’s instructions. The cDNA was synthesized from total RNA using the RevertAid First Strand cDNA Synthesis kit (Thermo Fisher Scientific, Inc.). The quantitative PCR (qPCR) of cDNA was performed using PowerUp SYBR Green Master Mix (Thermo Fisher, Waltham, MA) on a CFX96 Touch Real-Time PCR Detection System (Bio-Rad Laboratories, Hercules, CA, USA). Amplification was performed at 50 °C for 2 min, 95 °C for 2 min, followed by 50 cycles at 95 °C for 15 s and 60 °C for 1 min. The primer sequences are listed in Table 1.

### 2.7. Western Blotting

Western blotting was performed as previously described [1,24]. Briefly, lysate samples were resolved by 10% sodium dodecyl sulfate-polyacrylamide gel electrophoresis (SDS-PAGE) and transferred to a nitrocellulose membrane (LI-COR) for immunoblotting. The membrane was probed with mouse anti-nSMase2 (1:250, Santa Cruz Biotechnology, Inc.) and mouse anti-β-Actin (1:5000, Novus Biologicals, Lincoln, NE, USA) at 4 °C overnight. After washing with 1 × TBST, the membrane was incubated with IRDye 800 goat anti-mouse IgG (1:10,000, LI-COR Biosciences) for 1 h at room temperature. Detection was performed using an Odyssey infrared imager (LI-COR Biosciences, Lincoln, NE, USA).

### 2.8. Cell Apoptosis Assay

We used an in vitro hypoxia model to simulate cardiac ischemia. Mouse cardiomyocyte cells (HL-1) cultured in BD GasPak Pouches (BD Biosciences, Cockeysville, MD, USA) were subjected to hypoxia (0.1% oxygen) for 16 h [25]. HL-1 cell apoptosis was detected using the DeadEnd™ Colorimetric TUNEL System. The TUNEL assay was performed according to the manufacturer’s instructions with minor modifications. The TUNEL positivity of cells was quantified by fluorescence-activated cell sorting (FACS) analysis using a NovoCyte Quanteon flow cytometer (Agilent, Santa Clara, CA, USA). We first compared the apoptosis of hypoxia-exposed HL-1 cells incubated with CM from electrically stimulated C-MSC (C-MSC^ELSM^) or control C-MSC (C-MSC^Ctrl^). We also compare the apoptosis of hypoxia-exposed HL-1 cells incubated with EV-depleted (EV-del) CM from C-MSC^ELSM;EV-del^ and C-MSC^Ctrl;EV-del^. To interrogate the functional role of nSMase2 in EV-mediated cytoprotection, we compared apoptosis in HL-1 cells incubated with CM from C-MSC treated with siRNA to knock down nSMase2 versus control siRNA.

### 2.9. Statistical Analysis

All data were expressed as the mean ± SEM of at least three independent experiments for each group of different biological samples. An unpaired student’s *t*-test (GraphPad Prism version 9.41) was used to compare two groups. One-way ANOVA followed by Tukey’s post-hoc tests was used for comparisons between more than two experimental groups. Values of *p* < 0.05 were considered statistically significant.

## 3. Results

### 3.1. ELSM Stimulates EV Secretion of C-MSC

**Figure 1:**
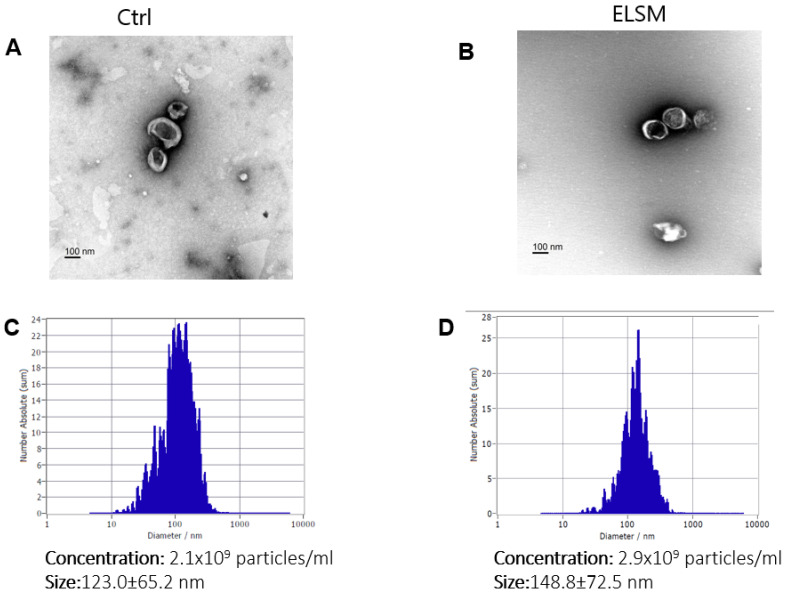
Characterization of EVs from C-MSC^Ctrl^ and C-MSC^ELSM^. (**A**,**B**) Transmission electron micrograph images of EVs from C-MSC. Scale bar = 100 nm. (**C**,**D**) nanoparticle tracking analysis using ZetaView^®^ Particle Tracking Analyzer to measure the size and number of C-MSC-derived EVs with or without ELSM.

Morphological analysis (electron microscopy) of pellets collected from conditioned medium of C-MSC treated without and with ELSM showed the typical appearance of extracellular vesicles (Figure 1A,B). The size of EVs was determined using ZetaView^®^, with EVs from C-MSC^Ctrl^ consisting of particles with an average diameter of 123.0 ± 65 nm, whereas EVs from C-MSC^ELSM^ had an average diameter of 148.8 ± 72 nm. The concentration of EVs in CM from C-MSC^Ctrl^ was 2.1 × 10^9^ particles/Ml, whereas the concentration of EVs from C-MSC^ELSM^ was 2.9 × 10^9^ particles/mL, indicating that ELSM treatment increased EV secretion from C-MSC by approximately 38% (Figure 1C,D).

### 3.2. EVs from ELSM-Treated C-MSC Protect Cardiomyocytes against Hypoxia-Induced Apoptosis

**Figure 2:**
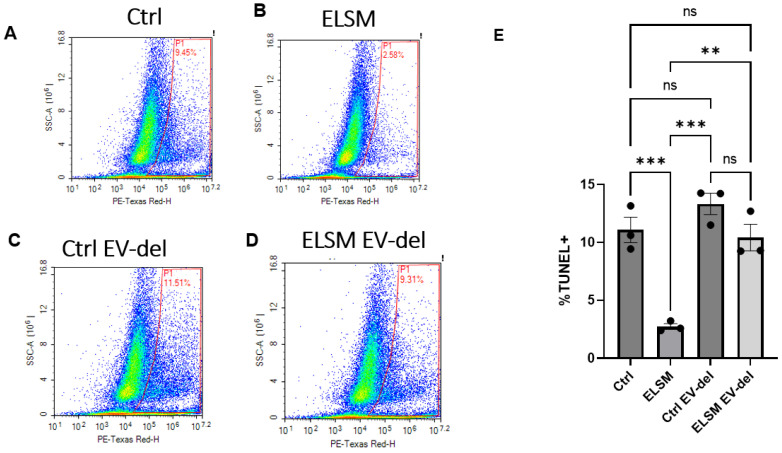
HL-1 cardiomyocytes treated with CM from ELSM-treated C-MSC showed reduced hypoxia-induced apoptosis, as determined by TUNEL staining and flow cytometry. (**A**,**B**) percentage of TUNEL positivity in HL-1 cells treated with CM from C-MSC^Ctrl^ or C-MSC^ELSM^; (**C**,**D**) percentage of TUNEL positivity in HL-1 cells treated with EV-depleted CM from C-MSC^Ctrl^ or C-MSC^ELSM^; (**E**) statistical analysis of the percentage of TUNEL-positive cardiomyocytes in the 4 groups (ns not significant; ** *p* < 0.01, *** *p* < 0.001, *n* = 3).

We previously reported that C-MSC-derived EVs protected cardiomyocytes from acute myocardial ischemia/reperfusion injury [7]. To investigate whether the ELSM-induced increase in EV secretion might enhance the cardioprotective effects, we incubated HL-1 cardiomyocytes with CM from C-MSC^ELSM^ or C-MSC^Ctrl^ under hypoxic stress for 16 h. The TUNEL assay showed significantly reduced apoptosis in HL-1 cells treated with CM from MSC^ELSM^ as compared with C-MSC^Ctrl^ (Figure 2A,B,E). However, when we treated HL-1 cardiomyocytes with EV-depleted CM, the differences in apoptosis between MSC^ELSM^ and C-MSC^Ctrl^ were abolished, suggesting that the ELSM-induced, enhanced cardioprotective effect was EV dependent (Figure 2C–E).

### 3.3. ELSM Increases nSMase2 Expression in C-MSC

**Figure 3:**
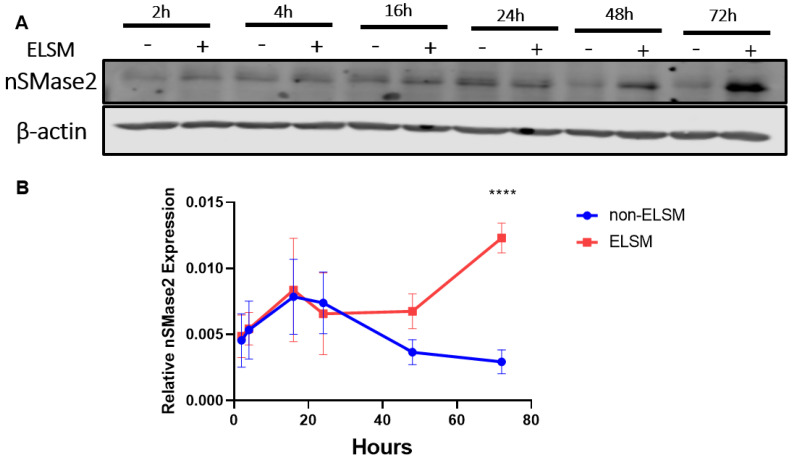
nSMase2 protein levels were determined by Western blot after treatment with or without ELSM for the indicated times. (**A**,**B**) Western blot analysis of nSMase2 expression levels in C-MSC; β-actin protein levels were used as loading control (**** *p* < 0.0001, *n* = 3).

NSMase2 is a key regulator that catalyzes ceramide formation, a critical step in extracellular vesicle biogenesis and release [21,26]. To determine whether ELSM treatment increases nSMase2 expression in C-MSC, we measured the time course of changes in nSMase2 protein levels after ELSM treatment. Western blotting analysis revealed that the expression of nSMase2 was similar in C-MSC^ELSM^ and C-MSC^Ctrl^ over the first 24 h. However, at 48 h, expression levels began to diverge, and at 72 h, there was a significant increase in nSMase2 protein levels in C-MSC^ELSM^ compared with C-MSC^Ctrl^ (Figure 3A,B).

### 3.4. ELSM-Induced EV Secretion Is Dependent on nSMase2

**Figure 4:**
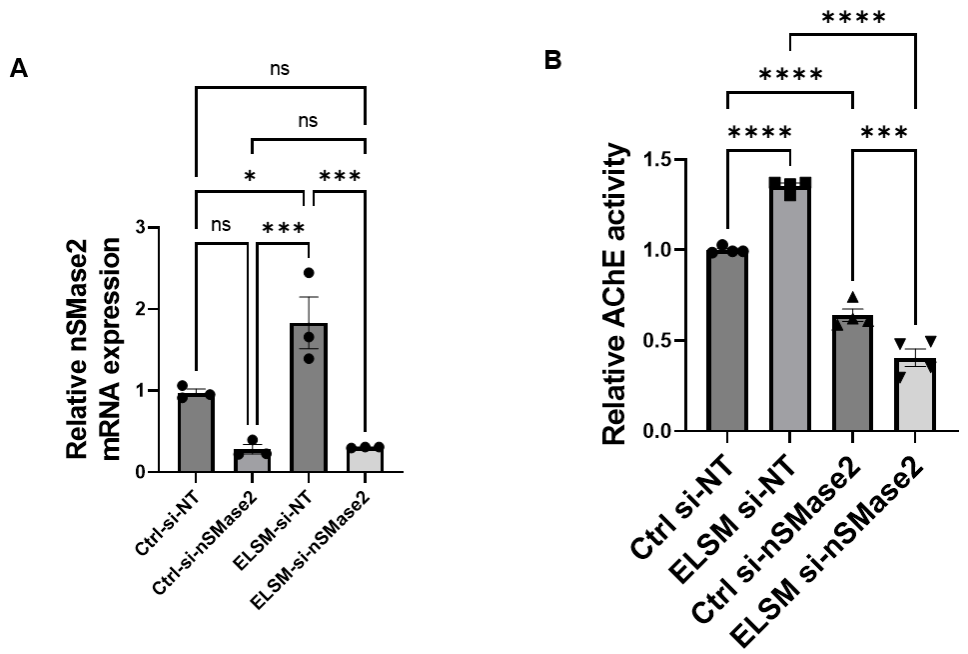
Knockdown of nSMase2 inhibits ELSM-induced EV release from C-MSC. (**A**) qRT-PCR quantification of nSMase2 mRNA levels (normalized to β-actin levels) in C-MSC transfected with nSMase2 siRNA (si-nSMase2) versus si-NT with/without ELSM treatment (ns not significant; * *p* < 0.05, *** *p* < 0.001, *n* = 3). (**B**) Quantification of relative AChE activity (normalized to protein concentration and then normalizing to the Ctrl si-NC group) in EVs from si-NT and si-nSMase2-transfected C-MSC with/without ELSM treatment (*** *p* < 0.001, **** *p* < 0.0001, *n* = 4).

To determine whether increased EV secretion in C-MSC^ELSM^ was mediated by increased expression of nSMase2 in C-MSC^ELSM^, we knocked down nSMase2 expression in C-MSCs with siRNAs targeting nSMase2. We measured nSMase2 mRNA levels in C-MSC by qRT-PCR, which confirmed that nSMase2 mRNA was decreased by about 70% in si-nSMase2-treated C-MSC^Ctrl^ compared to si-NT-treated C-MSC^Ctrl^. Additionally, there was ~80% downregulation of nSMase2 mRNA in si-nSMase2-treated C-MSC^ELSM^ compared to si-NT-treated C-MSC^ELSM^, indicating the efficiency of si-nSMase2 in knocking down nSMase2 in C-MSC treated with or without ELSM (Figure 4A).

To determine whether ELSM-stimulated EV release is nSMase2-dependent, we quantified acetylcholine esterase (AChE) activity in EV in CM from C-MSC [27]. In control cells, ELSM increased AChE activity in EV, consistent with a stimulatory effect on EV secretion (Figure 4B). Interestingly, nSMase2 knockdown completely abolished the ability of ELSM to stimulate EV secretion, indicating that the ELSM-induced increase in EV release from C-MSCs is dependent on nSMase2 (Figure 4B).

### 3.5. nSMase2 Is Directly Involved in the Modulation of Apoptosis in HL-1 Cells by the CM from ELSM-Treated C-MSC

**Figure 5:**
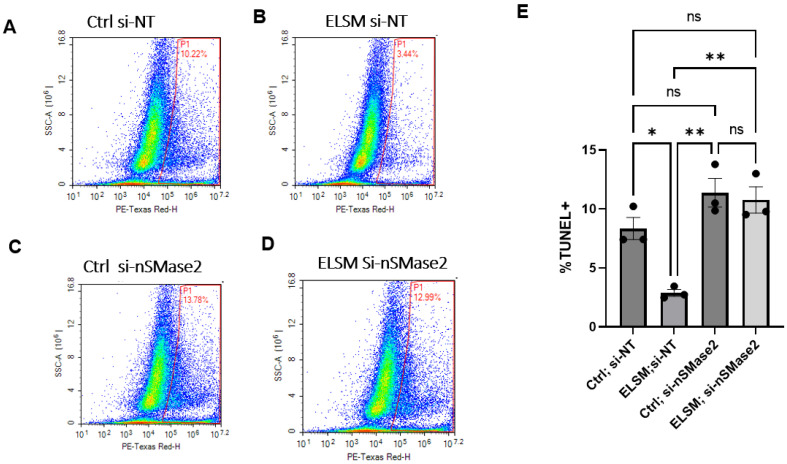
Reduced apoptosis in HL-1 cardiomyocytes treated with CM from ELSM-treated C-MSC is dependent on nSMase2. (**A**–**D**) Percentage of TUNEL+ cardiomyocytes treated with CM from si-NT transfected C-MSC without ELSM, si-NT transfected C-MSC with ELSM, si-nSMase2 transfected C-MSC without ELSM, and si-nSMase2 transfected C-MSC with ELSM; (**E**) statistical analysis of the percentage of TUNEL+ cardiomyocytes in the 4 groups (ns not significant; * *p* < 0.05, ** *p* < 0.01, *n* = 3).

We observed that CM from C-MSC^ELSM^ inhibited hypoxia-induced apoptosis in cardiomyocytes in a manner that was dependent on EV secretion. To investigate whether increased SMase2 expression is responsible for the protective effects of CM from C-MSC^ELSM^ against apoptosis in cardiomyocytes, we compared hypoxia-induced apoptosis in HL-1 cardiomyocytes treated with CM from C-MSC^Ctrl si-NT^, C-MSC^Ctrl si-nSMase2^, C-MSC^ELSM si-NT^, and C-MSC^ELSM si-nSMase2^. Importantly, there was no significant difference in apoptosis between the C-MSC^Ctrl si-nSMase2^ and C-MSC^ELSM si-nSMase2^ groups, suggesting that nSMase2 is directly involved in the modulation of apoptosis in HL-1 cells by the CM from ELSM-treated C-MSC (Figure 5A–E).

## 4. Discussion

**Figure 6:**
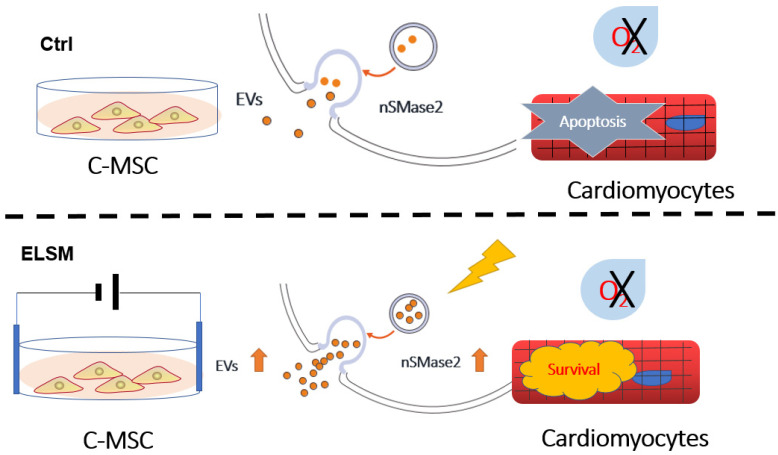
Schematic overview of proposed the mechanisms of ELSM-induced cardiomyocyte protection. ELSM increases expression of nSMase2, leading to increased EV secretion, which protects cardiomyocytes from hypoxia-induced apoptosis.

Electrical stimulation associated with CRT has been shown to be an effective treatment for patients with congestive heart failure (CHF; LVEF ≤ 35%), in part by producing favorable effects on cardiac metabolism. However, most patients with heart failure are not candidates for CRT, and for some of these patients, cardiac contractility modulation (CCM) is another therapeutic option involving ELSM. Indeed, a recent randomized controlled trial demonstrated that CCM improved exercise tolerance and quality of life in specific groups of heart failure patients, resulting in fewer heart failure hospitalizations [28]. Our findings indicate that ELSM increases EV secretion from C-MSC, thereby enhancing the protective effects of C-MSC-derived CM against hypoxia-induced apoptosis in cardiomyocytes. The ELSM-mediated secretion and subsequent anti-apoptotic effects of EV were dependent on nSMase2. These findings may have important implications for understanding the beneficial effects of CRT (Figure 6).

ELSM has been found to enhance cell activities, including proliferation, growth, migration, and differentiation, showing an important potential to manipulate cellular activity under normal and pathological conditions [29]. In addition, ELSM has been developed as a biophysical environmental cue for organized tissue engineering strategies with various electric field stimulation systems, which have been shown to affect the morphology, orientation, migration, and phenotype of several different cell types [30]. A recent study demonstrated that electric stimulation increased the expression of Ca^2+^ handling proteins and promoted the maturation of structural, mechanical, and firing properties of human-induced pluripotent stem cell-derived cardiac tissue [31].

EVs play important roles in cell-to-cell communication in tissue homeostasis and disease conditions [32]. A recent study reported that ELSM enhances neuronal cell activity mediated by EVs derived from Schwann cells [33], but how EV-based C-MSC-cardiomyocyte interactions are modulated by ELSM is poorly understood. C-MSCs reside in the cardiac niche and play a supportive role in the regulation of physiological cardiac function. They also play a crucial role in promoting cardiac repair and regeneration by differentiation and/or paracrine actions to stimulate cardiomyocyte proliferation, activation of resident cardiac stem cells, and induction of cardiac angiogenesis in pathological states, such as myocardial infarction [34]. We used a co-culture model to assess the effects of C-MSCs on HL-1 cardiomyocytes. There are two methods for co-culturing C-MSCs with cardiomyocytes: (1) direct co-culture of C-MSCs (or CM secreted from C-MSCs) with cardiomyocytes at a specific ratio, such as 1:1; and (2) indirect co-culture, where C-MSCs and cardiomyocytes are cultured in the same well but separated by a membrane that prevents cells from passing through [35]. With the first method, it is challenging to differentiate the effects of direct cell-cell contact from indirect paracrine effects. In the second method, the distance between the cells can impact the travel of vesicles that protect cardiomyocytes. In our study, we directly applied the CM from C-MSCs stimulated with ELSM or control C-MSCs to cardiomyocytes. This approach did not allow us to estimate the distance that the vesicles might travel, either in vitro or in vivo, to elicit paracrine effects.

Our findings indicate that ELSM increases EV secretion from C-MSC. Enhanced EV secretion is an adaptive cellular response to stress, such as hypoxia and inflammation. Hypoxia exposure has been reported to promote EV secretion from a variety of cells, including endothelial cells, cardiac progenitor cells [36], cardiomyocytes [37], and renal proximal tubule cells [38]. Zhang M. et al. [39] reported that CD63, the biomarker of EVs, was highly expressed in the intima of pulmonary arteries of hypoxic mice in a time-dependent manner compared to the normoxic mice, consistent with hypoxia-induced EV secretion. Interestingly, Panigrahi GK et al. [40] reported that hypoxia-induced EV secretion is more prominent in prostate cancer cells isolated from African Americans; moreover, lactate content was higher in EVs secreted under hypoxia, suggesting that prostate cancer cells secreted more EVs as a survival mechanism to remove metabolic waste. Tumor necrosis factor-α (TNF-α) [41], an inflammatory mediator, was reported to increase EV secretion from retinal pigment epithelial cells [42]. Moreover, oxidative stress can also regulate EV secretion. Wang R et al. [43] reported that 1 μM H_2_O_2_ significantly induced the migration of normal lens epithelial cells (LECs) by EV secretion. In this study, we found that ELSM-induced EV release from C-MSC. We did not perform studies testing the effects of ELSM on the heart in vivo. However, Klein et al. [44] found that low-frequency ELSM applied to the gastrocnemius muscle increased circulating EV abundance and altered EV-associated microRNA expression.

Local and transient ELSM was reported to promote the secretion of EVs carrying transcribed mRNA and targeting peptides. To determine whether increased EV secretion protects cardiomyocytes from hypoxia-induced apoptosis, we treated cardiomyocytes under hypoxic stress with CM from C-MSC^ELSM^, which confirmed the beneficial paracrine effects of C-MSC^ELSM^, consistent with prior studies in other cell types [19]. To further determine whether EVs were responsible for the cytoprotective effects of CM from ELSM-treated C-MSC, we removed EVs from CM by ultracentrifugation and found that the anti-apoptotic effect of C-MSC^ELSM^-CM was significantly diminished, indicating that the enhanced cardioprotective effects of C-MSC^ELSM^ were indeed EV dependent.

NSMase2 and ceramide are key regulators of biogenesis and the shedding of EVs in response to external stress [20]. The budding of EVs has been shown to depend on the conversion of sphingolipids to ceramides by nSMase2 [45], and blocking nSMase2 activity has been reported to reduce EV synthesis and secretion [26,46,47,48,49]. Recent studies have shown that the hypoxia-induced increase in EV secretion is dependent on hypoxia-inducible factor-1 (HIF-1) [38]. The capacity of MSC-derived EVs to improve cardiac function is enhanced by nSMase2-dependent transfer of microRNA-210 under hypoxic treatment [26]. Interestingly, nSMase2 was also reported to regulate the entry of miR-10b into EVs derived from metastatic breast cancer cells [50,51]. However, the underlying molecular mechanisms by which ELSM enhances the release of EVs from C-MSC remain unknown. We measured nSMase2 protein levels in response to ELSM in an immunoblotting time course study, which showed that nSMase2 levels increased over 48–72 h compared with C-MSC^Ctrl^. We also found that EV secretion in ELSM-enhanced C-MSC was abolished by nSMase2 knockdown, suggesting a key role for nSMase2 in ELSM-induced EV secretion. Importantly, knockdown of nSMase2 abolished the protective effects of CM from ELSM-treated C-MSC, suggesting that nSMase2 is indirectly involved in the modulation of apoptosis in HL-1 cells by regulating EV secretion from ELSM-treated C-MSCs.

ELSM can also change the energy metabolism of cells. For example, ELSM was reported to alter fatty acid metabolism in isolated skeletal muscle, along with exogenous free fatty acid (FFA) oxidation, exogenous FFA incorporation into intracellular triacylglycerol (TG), and intracellular TG content in the isolated muscle fibers [52]. ELSM was also reported to increase glucose metabolism in neurons detected by micro-PET imaging of FDG uptake [53]. Global analysis of the transcriptome and proteome demonstrated that ELSM significantly regulated proteins and genes enriched in biological processes related to glycolytic pathways, fatty acid oxidation, oxidative phosphorylation, as well as autophagy/mitophagy and oxidative stress, in human myotubes [54].

Cellular stresses, such as hypoxia and inflammation, have been reported to alter the content of EVs [55]. ELSM treatment has the potential to affect DNAs, RNAs (e.g., long noncoding RNAs, mRNAs, microRNAs, etc.), lipids, and proteins (e.g., enzymes, receptors) contained in EVs from C-MSC. Previous studies have shown that ELSM altered cellular transcriptional regulation, inducing transcription factors such as NFAT3, GATA4, NRF-1 (nuclear respiratory factor 1), c-Jun, and cytochrome C, leading to increased growth and maturation of cardiomyocytes, mitochondrial proliferation, and progenitor cell differentiation [56,57,58]. Yu B et al. [59] found that EVs from GATA4-overexpressing MSC carry high concentrations of microRNAs (e.g., miR-19a, miR-451, and miR-221) and growth factors (e.g., IGF-1). Hao C. et al. [60] also demonstrated that overexpression of GATA4 enhanced the protective effect of cardiac colony-forming unit fibroblast-derived EVs against myocardial ischemic injury by transferring miR221, which inhibited PTEN expression by activating the phosphatidylinositol 3-kinase (PI3K)/AKT signaling pathway. NRF-1 is a transcription factor that regulates the expression of mitochondrial transcription factor A (TFAM), a transcription factor required to initiate the transcription and duplication of mtDNA and thus mitochondrial synthesis [61]. A recent report showed that the expression of NRF-1 and TFAM was significantly decreased after hepatic ischemia-reperfusion injury subsequent to hepatectomy, but EVs derived from adipose-derived MSC had the ability to enhance mitochondrial biogenesis by increasing NRF-1 and TFAM expression [62]. ELSM treatment was also reported to increase the mRNA level of c-FOS/ERK1/2 in electrically stimulated cells [63], c-FOS is a transcription factor for ERK1/2. Epigenetic regulation might also be involved in ELSM-induced gene regulation. Klymenk O. et al. [64] reported that ELSM-induced interleukin 6 (IL6) transcription was enhanced in histone deacetylase 5(HDAC5)-deficient C2C12 myotubes. Recent studies have shown that EV-derived microRNAs contribute to cardioprotection by acting as anti-apoptotic mediators [65,66]. Electrical stimulation acupuncture has also been reported to increase renal blood flow via exosome-delivered miR-181 [44].

According to a recent study, inhibiting the secretion of EVs using the nSMase 2 inhibitor GW4869 resulted in a significant increase in cell senescence. This was demonstrated by showing an increase in the expression of several senescence-associated markers, including γH2AX, senescence-associated β-galactosidase (SA-β-gal), IL6, MMP13, and P16. Additionally, treating mice with GW4829 systemically for two months led to accelerated aging, as evidenced by increased staining of γH2AX and SA-β-gal in the liver, spleen, lung, and kidney. Furthermore, inhibiting EV release through knocking out the small GTPase Rab27a also accelerated senescence in various cells and mice [67]. Mitochondrial function, particularly electron transport chain (ETC) activity, is known to be important in inducing premature senescence [68]. Inhibition of EV secretion through knockdown of the small GTPase Rab27b was found to impair oxidative phosphorylation in cardiac mesenchymal stem cells and to down-regulate the expression of key genes involved in fatty acid β-oxidation, TCA, and ETC [1]. This suggests that EV secretion may have a beneficial effect against senescence by regulating metabolism.

## 5. Limitations and Future Direction

We presume that the protective effects of the conditioned medium are derived from both the quantity and quality of EVs. While it is straightforward to quantify EVs present in the conditioned medium, identifying the specific components (proteins, lipids, or RNAs) responsible for these beneficial effects poses a significant challenge. Future studies (i.e., mass spectrometry, lipidomic profiling) are required to explore the molecular content of EVs from ELSM-treated C-MSC. This will enable identification of specific mechanism of apoptosis protection by ELSM (i.e., reduction in ceramide levels).

In this study, we used a previously developed ELSM protocol [18], and future studies are needed to investigate the combination of pacing dose and pacing duration to obtain optimal pacing output, which may provide useful information for the benefit of multi-electrode pacing across the left ventricle.

Meraviglia V et al. [69] reported that ELSM significantly reduced the expression of connexin43 (Cx43) by increasing the acetylation of Cx43 on HL-1 cardiomyocytes at 0.5 Hz for 24 h, suggesting that ELSM may reduce the expression of Cx43 by affecting the protein level of Cx43 and reducing cardiomyocyte-to-cardiomyocyte communication. Another report suggested that Cx43 expression was highly sensitive to ELSM in a frequency-dependent manner that high stimulation frequency leads to a substantial reduction in global Cx43 expression in murine-induced pluripotent stem cell-derived cardiomyocytes by promoting the expression of microRNA-1, which targets Cx43 [70]. It would be interesting to see whether ELSM inhibits Cx43-dependent cell-cell communications and enhances EV-mediated intracellular communications.

## Figures and Tables

**Table 1:** Primer list.

Gene	Sequence (5′-3′)
β-Actin FWD (mouse)	AGAGCATAGCCCTCGTAGAT
β-Actin REV (mouse)	GCTGTGCTGTCCCTGTATG
nSMase2 FWD (mouse)	CTACATCGATTCTCCCACCAAC
nSMase2 REV (mouse)	CACAGAGGCTGTCCTCTTAATG

## Data Availability

The data presented in this study are available on request from the corresponding author.
